# The relationship between orthorexia nervosa symptomatology and body image attitudes and distortion

**DOI:** 10.1038/s41598-021-92569-2

**Published:** 2021-06-25

**Authors:** Adrianne Pauzé, Marie-Pier Plouffe-Demers, Daniel Fiset, Dave Saint-Amour, Caroline Cyr, Caroline Blais

**Affiliations:** 1 Département de Psychoéducation et de Psychologie, Université du Québec en Outaouais, Succursale Hull, C.P. 1250, Gatineau, QC J8X 3X7 Canada; 2 Département de Psychologie, Université du Québec à Montréal, Succursale Centre-Ville, C.P. 8888, Montreal, QC H3C 3P8 Canada

**Keywords:** Perception, Psychology, Psychiatric disorders

## Abstract

Orthorexia Nervosa (ON), a condition characterized by a fixation on healthy eating, still does not conform to any consensus concerning diagnostic criteria, notably in regard to a possible body image component. This study investigated the relationship between ON symptomatology, measured with the Eating Habit Questionnaire, and body image attitudes and body image distortion in a non-clinical sample. Explicit body image attitudes and distortion were measured using the Multidimensional Body-Self Relations Questionnaire. Implicit body image attitudes and distortion were assessed using the reverse correlation technique. Correlational analyses showed that ON is associated with both explicit and implicit attitudes and distortion toward body image. More precisely, multivariate analyses combining various body image components showed that ON is mostly associated with explicit overweight preoccupation, explicit investment in physical health and leading a healthy lifestyle, and implicit muscularity distortion. These findings suggest that ON symptomatology is positively associated with body image attitudes and distortion in a non-clinical sample. However, further studies should be conducted to better understand how ON symptomatology relates to body image, especially among clinical samples.

## Introduction

In our everyday life, we are increasingly exposed to various messages about food and nutrition. This growing interest toward nutrition in general, and healthy eating more specifically, is likely associated with positive impacts for society, notably because of its association with physical health^1,2^. However, it also appears to be simultaneous with the emergence of individuals displaying extreme concern for healthy eating^3^.

Such individuals showing an excessive preoccupation, or fixation, with eating food believed to be healthy are described as suffering from “Orthorexia Nervosa” (ON), a term that was first introduced by Steven Bratman^4^. According to his observations, ON would stem from a desire to become more healthy or to treat illness, rather than to lose weight. ON would involve excessive preoccupation with food considered to be healthy, excessive time dedicated to prepare and think about food, and adhesion to rigid dietary rules in regard to its quality. Furthermore, such adhesion would lead to a sense of superiority or, when inevitably breaking the self-imposed rules, would create intense guilt and adoption of more severe restrictions^3,4^. Research and case studies have shown that extreme concern for healthy eating may lead to significant physical, psychological and social impairments (see Dunn & Bratman^5^ and McComb & Mills^6^ for exhaustive literature reviews on ON). Although ON is not officially recognized as a mental health disorder^7,8^, some authors have suggested diagnostic criteria which go along with Bratman’s description^5,9,10^. However, no consensus has been reached, notably in regard to a potential body image disturbance component, namely how it would present itself, and whether it would be a core feature, an exclusion criteria, or an associated feature^11^.

Body image is commonly defined as a multidimensional psychological concept of experiencing one’s own body, including physical appearance, and encompassing attitudes and perceptions toward one’s body^12,13^. The attitudinal part of body image comprises subjective evaluation (body satisfaction or dissatisfaction and related evaluative beliefs), as well as cognitive, behavioral and emotional investment^12,14^. As for the perceptual component, it corresponds to the mental representation an individual has created of their own body or parts thereof^14^. Body image disturbance may encompass problems with the attitudinal component of body image, the perceptual component, or both, those dimensions being distinct from one another^15^. In terms of attitudes, body image disturbance may manifest itself, for example, through individuals dissatisfaction with their appearance, anxiety or shame about the body, distorted belief about appearance, excessive behavioral investment in appearance, or body avoidance^16^. Perceptual body image disturbance, commonly termed “body image distortion”, refers to a discrepancy between self-perceived appearance and actual appearance^14^.

A few prior studies have investigated the potential link between ON and body image attitudes. Bratman’s theory, which has been espoused by many authors^10,17,18^, suggests that ON excludes body image components^4^. Although current research does not allow to assert whether or not body image disturbance is a diagnostic feature of ON, results from the few studies investigating the association between ON and body image suggest that ON manifestations are related to some specific body image attitudes. As a matter of fact, ON was found to be positively associated with investment in and preoccupation toward appearance and fitness, overweight preoccupation, thinness and muscularity valorisation, and even exercise addiction ^19–25^. In terms of appearance satisfaction per se, while some data suggest that ON is associated with lower appearance satisfaction, other results suggest the opposite^19–21,26–29^. Despite those contradictory findings, the fact that many other attitudes pertaining to body image are also correlated with ON supports the idea that ON is associated with specific body image attitudes. However, methodological and psychometric limitations of those studies, which are further discussed below, and the paucity of research in this area highlight the need to keep investigating this question.

Besides, the current state of knowledge regarding the potential link between ON and the perceptual component of body image is even more sparse, although it similarly involves contradictory findings. While some studies have shown that ON is associated with higher self-perceived weight^19,30^, Oberle and Lipschuetz^31^ observed that ON is associated with higher self-perceived muscularity and with lower self-perceived body fat. Of note, all these studies relied on self-report measures; no objective body measures were included. Thus, it is impossible to infer from their results whether ON is associated with actual differences in body appearance, or with a body image distortion. One of the main aims of the present study was therefore to investigate the existence of a potential link between ON symptomatology and body image distortion.

As described above, while ON appears to be associated with specific body image attitudes, we do not know yet if it is also associated with body image distortion. Moreover, the aforementioned studies have limitations. First, they were almost all based on either the ORTO-15 questionnaire^32^ or the Bratman Orthorexia Test^3^, ON measures that have frequently been criticized in regard to their psychometric properties^33–35^. To address this issue, the present study used the Eating Habit Questionnaire (EHQ), a multidimensional and validated tool to better assess ON symptomatology^36–39^.

Second, body image assessments in ON literature have so far exclusively been based on self-report measures. Although such tools may cover a wide range of explicit body image attitudes, they might be biased by social desirability, and preclude a thorough comprehension of the body image concept in all its complexity. Thus, we also opted for a novel application of a technique called reverse correlation^40,41^ to implicitly measure body image dissatisfaction and distortion. This data-driven technique comes from the field of psychophysics and aims to reveal mental representations, which are influenced by perceptions^42,43^ and attitudes^44–46^. It has been successfully used in a number of perceptive domains ^40,41,44,47–51^.

**Figure 1:**
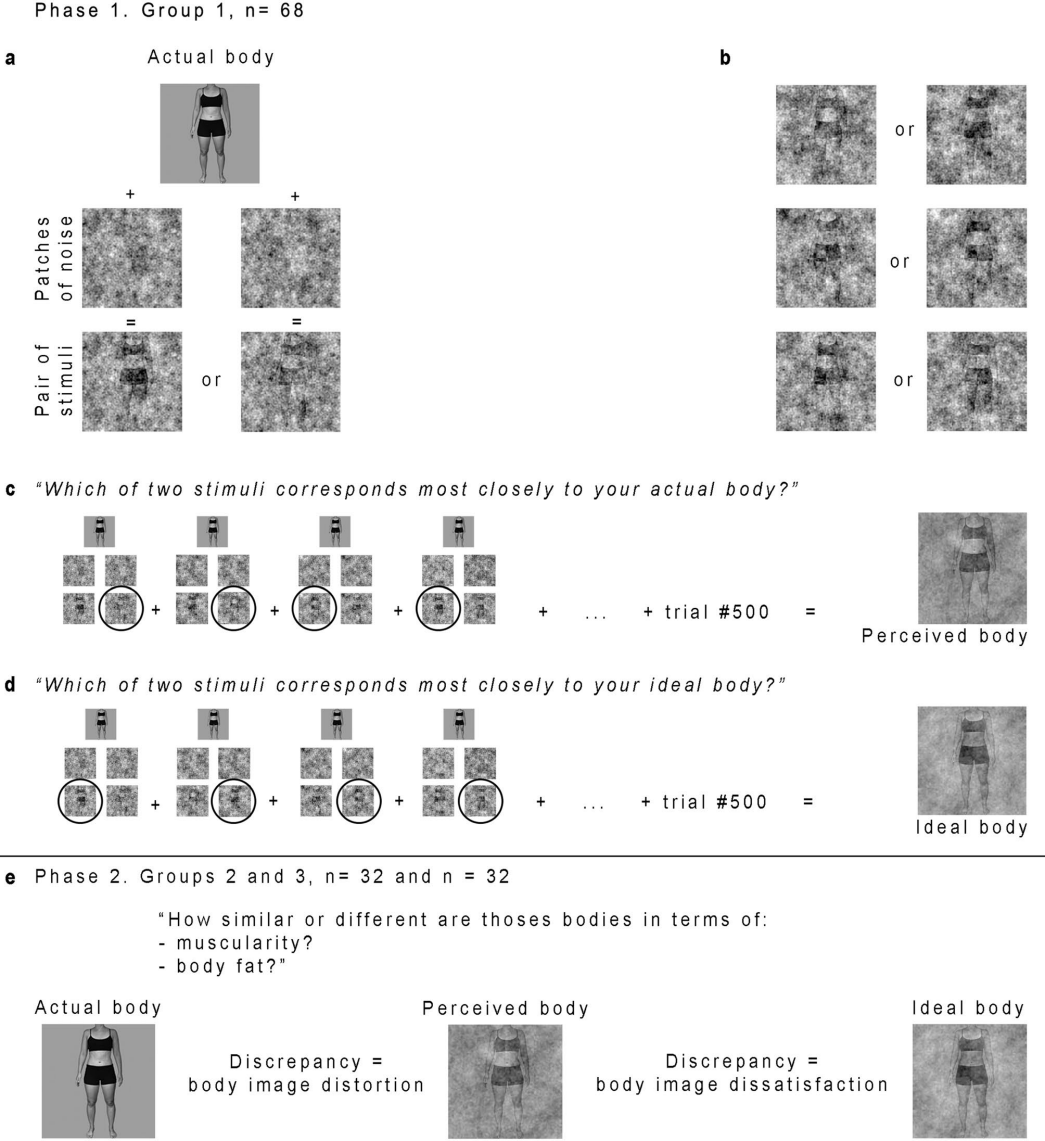
Reverse correlation method to measure body image dissatisfaction and distortion. **(a)** Example of the steps involved in the creation of a pair of stimuli in the image-construction phase using a participant’s photograph. **(b)** Examples of stimuli generated through the reverse correlation technique in the image-construction phase for this participant. Patches of visual noise added to her photograph randomly modulates her body’s appearance. **(c)** Example of trials in the first task (perceived body image). The two stimuli created on a given trial were presented side-by-side to the participant. The images selected by the participant in each of the 500 trials were averaged to produce her perceived body image representation. **(d)** Example of trials in the second task (ideal body image). **(e)** Example of comparisons by two new groups of independent participants during phase 2 to assess body image distortion and dissatisfaction of participants tested during phase 1.

In order to capture those representations, some visual noise is added to a single class of stimulus so as to randomly modulate its appearance. Participants then make a judgment on each of the variations (noisy stimuli) regarding its similarity to the mental representation of interest. After multiple trials, it becomes possible to infer what features of the added visual noise make a stimulus’ appearance closer to the participant’s mental representation. Mental representations of perceived and ideal body can thus be generated for each participant and used in order to measure both implicit body image distortion and body image dissatisfaction through visual comparisons between those images by an independent sample (see Fig. 1 for an overview of the method).

The reverse correlation technique presents notable advantages for the question addressed in the present study. For one, the mental representations extracted with this technique are unlikely to be tainted by social desirability, especially compared with self-report questionnaires. Most importantly, it does not rely on any assumptions regarding what defines body appearance. The participants, even if they were aware that they were looking at bodies, were not forced to think about a specific feature (e.g. size, fat, muscularity) or a specific part of their body; instead, they were completely free to rely on any dimension(s) to make judgments about self and ideal body appearance. As explained above, the technique consists in adding noise over a body, such that the noise modulates the body appearance. Noise is, by definition, random. Thus, the variations from one trial to another are very subtle; as a matter of fact, participants anecdotally frequently report having the impression of answering randomly on some trials. It is only when all of the trials are considered, in relation with the participants responses, that some features can stand out more clearly. For all of these reasons, we think that on a continuum ranging from completely implicit to completely explicit, reverse correlation falls closer to the implicit end of the continuum than most techniques used to assess body image. This idea is supported by studies in social psychology showing that the appearance of an individual’s given mental representation (e.g. how criminal-looking they imagine an other-ethnicity face) can be predicted by their score on an implicit measure of racism^44^.

The present study aimed to provide new information regarding the complex relationship between ON and the different components of body image in a non-clinical sample, with both explicit and implicit measures. Firstly, we tested whether or not ON symptomatology is associated with implicit body image attitudes. We hypothesized that ON is positively associated with implicit body image dissatisfaction. Secondly, we verified the generalizability of previously observed association between ON and explicit body image attitudes. We hypothesized that ON is positively associated with investment in and valorization of physical health, appearance, and physical fitness, as well as overweight preoccupation. We also hypothesized that ON is negatively associated with appearance satisfaction. Thirdly and fourthly, we investigated the potential link between ON symptomatology and, on the one hand, implicit body image distortion; on the other hand, explicit body image distortion. Because this is the first study to address specifically the question of an association between ON and body image distortion, we had no specific hypothesis with regard to the last two questions.

## Methods

Implicit body fat and muscularity dissatisfaction, as well as various explicit body image attitudes, were assessed in a non-clinical sample, namely adults recruited from the general population and varying in ON symptomatology. We also assessed implicit body fat and muscularity distortion, and explicit body size distortion. To improve this article’s legibility, those concepts are respectively referred to as *implicit body image attitudes, explicit body image attitudes, implicit body image distortion,* and *explicit body image distortion*. Since body image can encompass so many dimensions (e.g. emotions about specific body characteristics, body avoidance), we decided to limit ourselves to the aforementioned measures in the context of our research.

There were two phases to this study. In the first phase, defined as the *image-construction phase,* the reverse correlation technique was used in order to extract both perceived and ideal body representations for each participant. These mental representations reflect respectively the perceived and ideal bodies of the first sample group, termed the *image-construction sample*. In the second phase, participant photographs, as well as perceived and ideal body representations extracted during the first phase, were presented to a second sample of participants in order to obtain quantitative measures of their implicit body image dissatisfaction and distortion. We will refer to this second phase as the *image-comparison phase,* and participants will be termed the *image-comparison sample*.

This study was part of a larger research project in which other measures were collected (for a list, see Sect. 1 in Supplementary Material).

### Image-construction phase

#### Participants

The image-construction sample was composed of 68 adults from the general population (mean age of 26.9 ± 6.5; 59 women). The sample size was decided a priori in order to have a power of 0.8 and reveal an effect size of 0.3 in a correlation analysis (calculated with G*Power^52^). Moreover, that sample size is well aligned with a large part of the studies using reverse correlation techniques, with sample sizes ranging between 15 ^53,54^ and 60 participants^55–57^, although some studies have indeed used larger samples^46,58^.

One participant was of Black-African descent, was born in Democratic Republic of the Congo and immigrated to Canada when she was two years old. One was of Asian-descent, was born in China and immigrated to Canada when she was six months old. The remaining 66 participants were of White-European descent and were born in Canada or Western Europe.

Participants were recruited in two Canadian cities (Montréal and Gatineau) through advertisements on social media and mailing lists. All participants had normal or corrected-to-normal visual acuity, as confirmed upon their arrival using the Snellen scale^59^. They were all Francophones. All of them provided informed written consent prior to the experiment. As for photographs and images used in the figures of this article, informed written consent was obtained to publish the images in an online open access publication. The protocol of this experiment was approved by the Research Ethics Committee of Université du Québec en Outaouais and was conducted in accordance with the Code of Ethics of the World Medical Association (the Declaration of Helsinki).

#### Procedure

**Figure 2:**
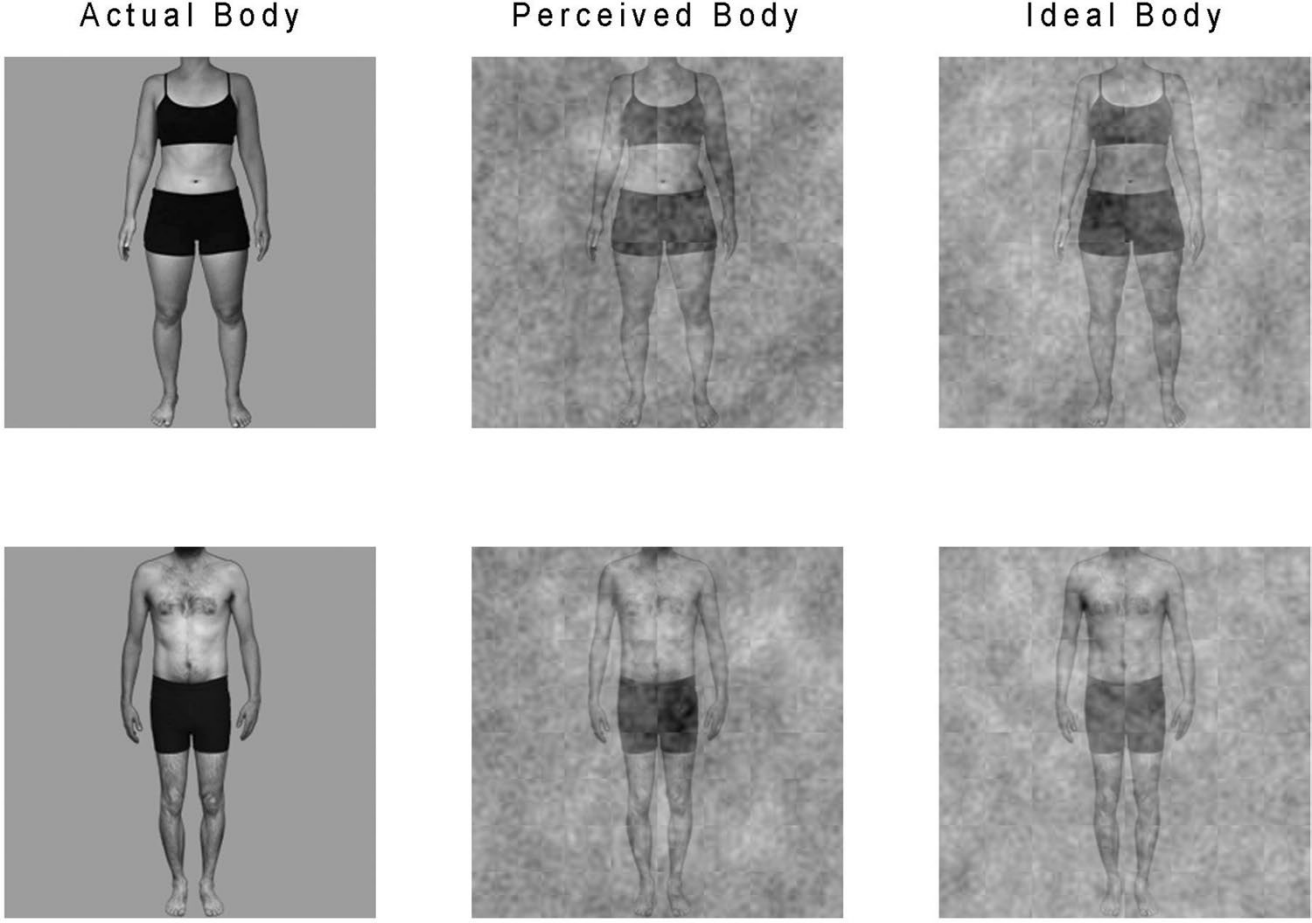
Examples of body images obtained in the image-construction phase. From left to right, a female and a male example of participants' actual bodies (participant’s photograph), perceived bodies (mental representation of their actual body) and ideal bodies (mental representation of their ideal body).

Participants were invited to the lab on two separate days. Upon their first visit, a photograph of their body was taken. Special clothes were provided in order to standardize, across diverse participants, the visibility of each body area (see Fig. 2 for an example of the clothes used for men and women). Clothing sizes ranging from X-small to 3X-large were available at the lab to account for differences in morphology in the population. The photographs taken on the first day were later used in the reverse correlation technique during the participants’ second visit.

Upon their second visit, participants completed the image-construction tasks using the reverse correlation technique, then the EHQ, and finally the MBSRQ. Financial compensation of $12 per hour was offered for participation.

#### Material

The reverse correlation technique and the questionnaires were completed on iMac computers. Calibrated LCD monitors with a spatial resolution of 1280 × 720 pixels and a refresh rate of 60 Hz were used. The experimental programs were written in MATLAB using functions from the Psychophysics toolbox^60–62^.

#### Measures

The EHQ^36^ is a 21-item self-report questionnaire designed to assess cognitions, feelings, and behaviors related to ON. Participants responded to each item using a 4-point Likert scale, where 1 corresponds to “Not at all true” and 4 corresponds to “Totally true”. The questionnaire is composed of three subscales: problems associated with healthy eating (12 items, e.g. “I am distracted by thoughts of eating healthily.”), healthy eating knowledge and behaviors (5 items, e.g. “I am more informed than others about healthy eating.”), and feeling positively about healthy eating (4 items, e.g. “I feel in control when I eat healthily.”). The higher the score is on the global scale or on each subscale, the more severe is ON symptomatology in a participant, with possible global scores ranging from 21 to 84. The three subscales respectively have an internal consistency of α = 0.90, α = 0.82 and α = 0.86, and a test–retest fidelity of *r* = 0.81, *r* = 0.81 and *r* = 0.72. No French version of this questionnaire previously existed, so we translated it using a back-translation procedure.

The Multidimensional Body-Self Relations Questionnaire (MBSRQ)^63,64^ is a 69-item self-report questionnaire divided into 10 independent subscales which measure different self-attitudinal aspects of body image, including evaluative, cognitive, and behavioral components. This questionnaire was used to assess explicit body image attitudes in regard to three somatic domains: Appearance, Fitness, and Health/Illness. Participants responded to each item using a 5-point Likert scale, with 20 items being reverse-scored. Each subscale’s score corresponds to the average of the relevant items after computing reverse-coded ones, resulting in possible scores ranging from 1 to 5. The internal consistency of the subscales ranges from 0.70 to 0.91 for males and from 0.73 to 0.90 for females^64^. No French version of this questionnaire previously existed, so we translated it using a back-translation procedure. A brief description of each subscale is provided here. Apperance Evaluation subscale evaluates feelings of attractiveness or unattractiveness. In other words, how satisfied or dissatisfied individuals are with their appearance. High scorers feel mostly positive and satisfied with their appearance, whereas low scorers feel generally unhappy with their appearance. Appearance Orientation subscale evaluates the extent to which one is invested in one’s own appearance. A higher score is typically obtained by individuals who place importance on how they look, pay attention to their appearance, and engage in extensive grooming behaviors. Fitness Evaluation subscale evaluates feelings of being physically fit or unfit. A higher score is typically obtained by individuals who feel physically fit or athletically active and competent. Fitness Orientation subscale evaluates how invested an individual is in being physically fit or athletically competent. The higher the score, the more invested an individual is. Health Evaluation subscale evaluates feelings of physical health and/or freedom from physical illness. A higher score indicates that the individual feels their body is in good health and that they do not experience bodily symptoms of illness or vulnerability to illness. Health Orientation subscale evaluates how invested an individual is in having a physically healthy lifestyle. A high score indicates that an individual is health-conscious and tries to lead a healthy lifestyle. Illness Orientation subscale evaluates reactivity to the idea of being or becoming ill. A high score indicates that an individual is attentive to possible symptoms of physical illness and is prepared to seek medical attention. Body area Satisfaction scale evaluates satisfaction with one’s own body areas and attributes. A high score is obtained by individuals who are generally satisfied with most areas of their bodies. Overweight Preoccupation scale assesses a construct combining self-reported measures of fat anxiety, weight vigilance, dieting, and eating restraint. Self-Classified Weight scale reflects how an individual perceives and labels their own weight, from very underweight to very overweight^64^. The reverse correlation technique was used to extract mental representations of the perceived and ideal bodies of each participant. The technique consists in adding visual noise to stimuli (in this case, each participant’s own photograph taken on their first visit) and asking participants to make judgments on the noisy stimuli. In the present study, participants completed two image-construction tasks, each comprising five blocks of 100 trials. During the first task, each participant needed to choose which of two noisy stimuli depicted most accurately their actual body. In the second task, they needed to choose which of two stimuli depicted most accurately their ideal body. During each trial, the two stimuli presented were generated by adding or subtracting a patch of sinusoidal white noise to the photograph of the participant (for more details on the noise properties, see Mangini & Biederman^41^). The body pictures were presented in greyscale, with a uniform grey background. The noise patches were randomly generated for each trial. The underlying idea of reverse correlation is that the visual noise randomly changes the appearance of the participant’s body photograph and makes it closer to or farther from their mental representation (see Fig. 1b for examples of how visual noise modulates body appearance). After several trials, it thus becomes possible to infer what properties in the visual noise are correlated with the participant’s percepts of their actual and ideal bodies. Figure 1a illustrates the steps for creating stimuli. If there is a systematicity in the kinds of alterations that lead a participant to perceive the displayed stimuli as being closer to their actual body (task 1; see Fig. 1c) or ideal body (task 2; see Fig. 1d), these can be captured by averaging all the noise patches that were part of the stimuli selected by the participant in each trial. The mental representation can then be visualized by adding this averaged noise to the base photograph that was used during the task, in the present case a photograph of each participant’s body. For each participant, mental representations were computed separately for each task, and subsequently added to the participant’s original photograph to generate the mental representations of both their perceived and ideal bodies. Participants' original photographs will be termed the *actual body*, whereas the two mental representations will respectively be termed the *perceived body* and *ideal body representations*. Examples of two participants' actual, perceived, and ideal bodies are provided in Fig. 2.

### Image-comparison phase

#### Participants

The image-comparison sample was composed of 64 adults (56 women; mean age of 25.5 ± 5.1). They were all of White-European descent and born in Canada or Western Europe. Recruitment procedures, inclusion criteria, and research ethics approval are the same as those in the image-construction phase. It is important to note that these participants were not part of the image-construction phase.

#### Procedure

Participants were invited to the lab once, where they completed the image-comparison task and the weight-classification task, in that order. Financial compensation of $12 per hour was offered for participation.

#### Material

Same as for the image-construction phase.

#### Measures

The image-comparison task served to quantify the discrepancy between actual, perceived, and ideal body representations obtained for each participant of the image-construction sample (see Fig. 1e). More specifically, participants were asked to compare pairs of stimuli on either muscularity (32 participants) or body fat (32 participants)*.* We selected muscularity and body fat because these dimensions overlap with previous literature on ON and body image^19–23,25,30,31^. However, participants in the image-construction phase were not asked to think about their body in terms of these two dimensions.

Two conditions were created. The first was designed to measure implicit body image dissatisfaction related to the dimensions of muscularity and body fat. For this first condition, a pair of stimuli composed of the perceived and ideal body representations of the participants from the image-construction sample was presented to the image-comparison sample. This comparison allowed us to quantify the distance between image-construction participants' perceived body and their ideal appearance. Large distances between the mental representations of the perceived and ideal body can be considered as a dissatisfaction index while small distances can be seen as a sign of body image satisfaction^14^. The second condition was designed to measure body image distortions, again as they relate to the dimensions of muscularity and fat. For this second condition, a pair of stimuli composed of the photograph of an individual from the image-construction sample and their perceived body representation were shown to the image-comparison sample. This comparison made it possible to evaluate the distance between image-construction participants' actual body and their perceived body. The overlap between the mental representation of participants' actual body and a true picture of their body reflects accuracy of one's self-perception, whereas distance is a sign of body image distortion^14,65^. The two conditions were interleaved during the experiment, and only differed in the nature of the images making up each pair.

**Figure 3:**
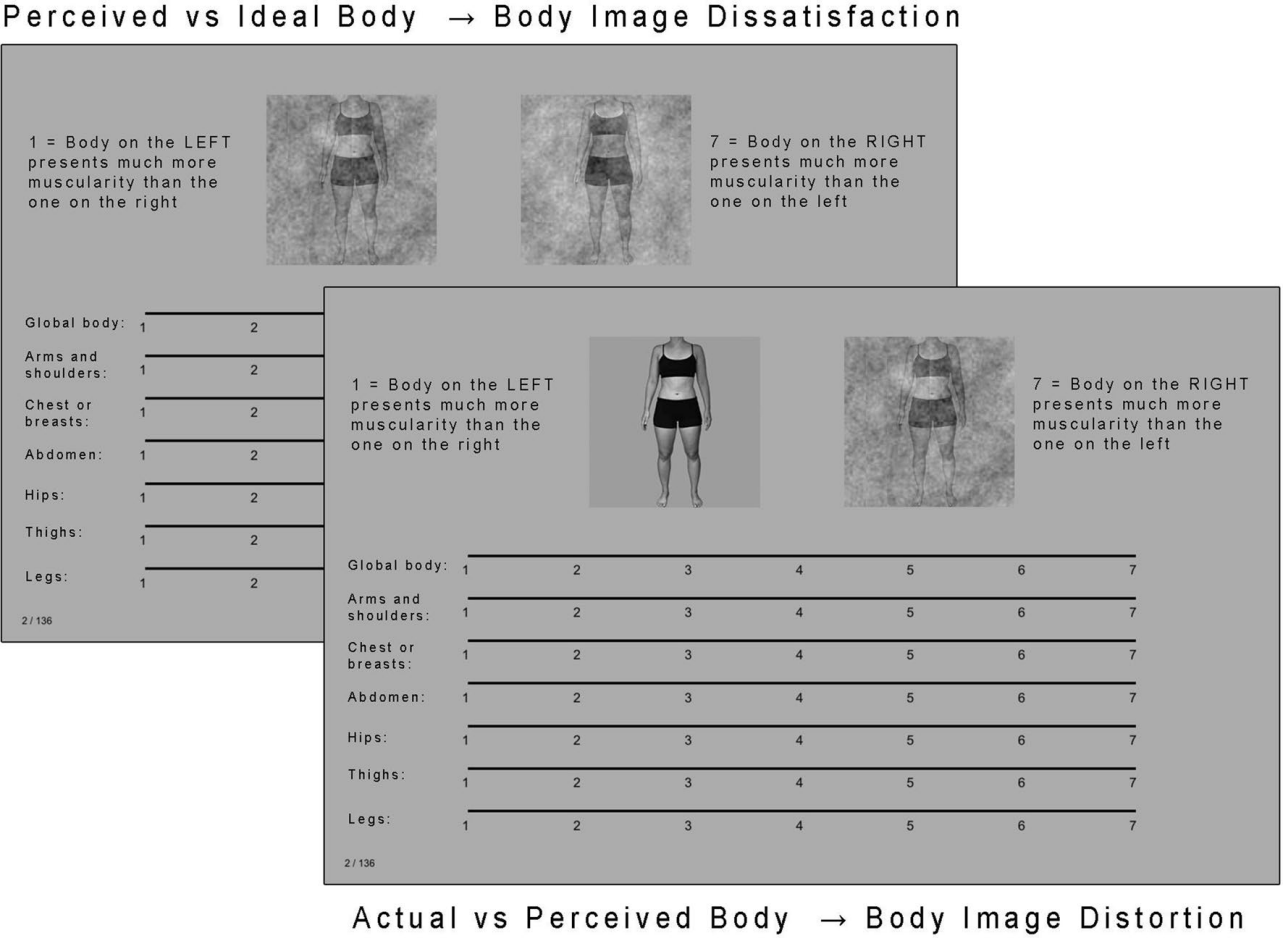
Examples of trials during the image-comparison phase. The left one represents the assessment of implicit muscularity dissatisfaction, and the right one represents the assessment of implicit muscularity distortion.

Each trial went as follows (see Fig. 3). Two images depicting the same individual, as described above, were presented on the screen. Seven-point Likert scales were displayed below the images. For participants comparing the images on the level of muscularity, the left side of the scales indicated “Body on the left presents much more muscularity than the one on the right”, and the right side indicated “Body on the right presents much more muscularity than the one on the left”. The middle point indicated “No difference between the 2 bodies regarding muscularity”. The same procedure was used for participants comparing the images with respect to body fat, but the word “muscularity” was replaced by “body fat”. In the first scale, participants were asked to compare the two images on their general level of muscularity or fat. The six remaining scales each represented a particular body area regarding which participants had to compare the two images: arms and shoulders, chest or breast, abdomen, hips, thighs, and legs. The position—left or right side of the screen of either actual, perceived, or ideal body representations—was determined randomly for each trial.

The weight classification task aimed to obtain an explicit measure of body size distortion, henceforth termed explicit body image distortion. The participants from the image-comparison sample were presented one more time with the original photographs of the 68 participants from the image-construction sample. They were asked to answer a modified version of the Self-Classified Weight subscale from the MBSRQ to describe the weight of the bodies presented. During each trial, a body photograph was presented, and participants were asked to evaluate the body weight of the photograph using a 5-point Likert scale ranging from “very underweight” to “very overweight”. The difference between the average score given to each body and the score each participant from the image-construction phase obtained on the Self-Classified Weight subscale resulted in a measure of explicit body image distortion. More specifically, a highly positive score was interpreted as an overestimation of body size, and a highly negative score reflected an underestimation of body size. A score of zero indicated that there was no difference between the perceived and actual body sizes.

### Statistical analyses

In order to examine the association between ON and various body image measures, we conducted Pearson's correlations. However, even if this study was conducted among participants recruited in a non-clinical population, a wide range of scores were expected, including extreme data. To make sure that the Pearson's correlations we obtained were not merely the results of such atypical data, Spearman's correlations were also computed and provided in the Supplementary Material (see Tables S1, S2 and S3).

We then proceeded to a stepwise regression to further explore the association between body image measures and ON. This analysis allowed to identify which body image components are the most related to ON symptomatology, and to document the relative contribution of various body image dimensions to ON.

Because of the disparity between the number of females (n = 59) and males (n = 9) in our sample, a non-parametric analysis was also conducted to verify the presence of a gender difference in ON distributions. Additionally, we assessed the stability of our results after adding or removing male participants from correlations and regression analyses.

Finally, Supplementary Material also includes descriptive statistics (see Tables S4 and S5), and correlational analyses between implicit and explicit measures of body image (see Table S6).

Prior to those analyses, preliminary data computing from the reverse correlation method was conducted. Scores given by participants during the image-comparison phase to evaluate body fat and muscularity discrepancies between actual, perceived and ideal bodies were arbitrarily coded from -3 to + 3. The underlying idea was that the middle point in the 7-point Likert scales, meaning that there is no difference between the two body images according to image-comparison participants, would represent a score of zero.

In terms of implicit body image dissatisfaction, depending on the dimension, a negative score indicated that image-comparison participants judged the ideal body generated in the image-construction phase as less muscular or fatter than the corresponding perceived body. By contrast, a positive score indicated that it was judged as more muscular or skinnier than the perceived body. A score of zero indicated no body fat or muscularity differences between the perceived and ideal bodies. For each pair of body representations, the average of the scores given by all participants in the image-comparison phase was then computed. These responses were also compiled for each of the seven body areas subscales, resulting in 14 scores per image-construction-phase participants. These scores provided insight as to whether or not they would ideally prefer their whole body, or one or many of the six specific body areas studied, to differ in terms of muscularity and/or body fat.

In terms of implicit body image distortion, depending on the dimension, negative scores indicated that image-comparison participants found the perceived body to be either more muscular or less fat than the actual body. By contrast, a positive score indicated that they found the perceived body to be either less muscular or fatter than the actual body. A score of zero indicated the absence of body fat or muscularity difference between the perceived body and the actual body. Again, those responses were also compiled for the subscales pertaining to each of the seven body areas, resulting in 14 scores per image-construction phase participant. These scores provided insight as to whether or not image-construction phase participants perceived their whole body, or any or many of the six specific body areas, as more or less muscular and/or fat than they actually are.

Additionally, a measure representing the most unsatisfactory body area was created, to take into account the possibility that individuals may differ in terms of which area is farther from their ideal body. If this were the case, when calculating correlations separately for each body area, a significant correlation between ON and body image dissatisfaction could go unnoticed. Our methodology took into consideration that a participant could either want more or less body fat and/or muscularity. Thus, for each participant, we selected the highest score for all areas combined, without regard to its mathematical sign (i.e. absolute score). We then retained this score, including its mathematical sign, as the value of each participant's most unsatisfactory area. In situations where a participant would have had their two absolute highest scores be tied, one being of positive value and the other being negative, that participant was removed from the analysis (maximum 4 times per analysis). In such cases, sample size is indicated in the results tables. This analysis was conducted for both body fat and muscularity. As was done for implicit body image attitudes, a measure representing the most distorted area was also created.

## Results

### ON and implicit body image attitudes (univariate analyses)

Results revealed significant positive Pearson's correlations between EHQ scores and body fat dissatisfaction for every area, with the exception of the legs (see Table 1). A significant positive relationship between EHQ scores and muscularity dissatisfaction regarding the chest or breast area was also observed, along with a marginally significant positive correlation between EHQ scores and hips’ muscularity dissatisfaction (*p* = 0.051). High positive reverse correlation scores are interpreted as a desire to be more muscular or less fat than the perceived body, depending on the dimension. Therefore, high EHQ scores were positively associated with a desire to have less body fat in one’s overall body, chest or breast area, abdomen area, hips area, the most unsatisfactory area, and be more muscular on the chest or breast and, possibly, the hips.

**Table 1:** Pearson's correlations (r) between EHQ scores and implicit body fat and muscularity dissatisfaction for various body areas.

	Body fat		Muscularity	
	*r*	*p*	*r*	*p*
Overall body	**0.314**	**0.009**	0.201	0.100
Arms and shoulders	**0.273**	**0.024**	0.212	0.083
Chest or breast	**0.287**	**0.018**	**0.266**	**0.028**
Abdomen	**0.301**	**0.013**	0.213	0.081
Hips	**0.269**	**0.027**	0.238	0.051
Thighs	**0.265**	**0.029**	0.078	0.527
Legs	0.126	0.307	0.151	0.218
Most unsatisfactory area	**0.286**^**a**^	**0.021**	0.214^b^	0.081

Significant results are indicated in bold characters.

^a^*n* = *65.*

^b^*n* = *67.*

Similar results were obtained when Spearman's correlations were conducted on EHQ scores and body fat dissatisfaction (see Table S1 in the Supplementary Material). Notably, Spearman's correlations conducted on EHQ and muscularity dissatisfaction revealed significant correlations for a higher number of body areas, namely for the overall body, arms and shoulders, chest or breast, hips, and the most unsatisfactory area.

### ON and explicit body image attitudes (univariate analyses)

The results showed a significant positive association between EHQ scores and Appearance Orientation, Fitness Orientation, Health Orientation, and Overweight Preoccupation from the MBSRQ (see Table 2). Although not statistically significant, a negative correlation near the significance threshold was observed between EHQ scores and Body Areas Satisfaction (p = 0.054).

**Table 2:** Pearson's correlations (r) between EHQ and MBSRQ subscales (explicit attitudes). Significant results are indicated in bold characters.

	EHQ	
	*r*	*p*
Appearance Evaluation	−0.205	0.093
Appearance Orientation	**0.299**	**0.013**
Fitness Evaluation	−0.084	0.495
Fitness Orientation	**0.339**	**0.005**
Health Evaluation	−0.042	0.735
Health Orientation	**0.367**	**0.002**
Illness Orientation	0.074	0.547
Body Areas Satisfaction	−0.235	0.054
Overweight Preoccupation	**0.569**	** < 0.001**
Self-Classified Weight	0.112	0.365

There was, however, no significant correlation between EHQ scores and Appearance Evaluation, Fitness Evaluation, Health Evaluation, Illness Orientation, and Self-Classified Weight.

Spearman's correlations yielded similar results (see Table S2 in Supplementary material).

### ON and implicit body image distortion (univariate analyses)

Pearson's correlations (see Table 3) and Spearman's correlations (see Table S3 in the Supplementary Material) revealed no significant correlation between EHQ scores and implicit body fat distortion. Pearson's correlation yielded nearly significant positive correlations between EHQ scores and implicit muscularity distortion regarding the abdomen (p = 0.051), and the arms and shoulders (p = 0.053). Spearman's correlation yielded a significant positive correlation between EHQ scores and abdomen muscularity distortion. Since positive reverse correlation scores are interpreted as an underestimation of perceived muscularity compared to the actual body, these results suggest that high EHQ scores were associated with an underestimation of abdomen muscularity. High EHQ scores were also potentially associated with an underestimation of arms’ and shoulders’ muscularity. However, these last data points must be interpreted cautiously since they did not reach the significance threshold.

**Table 3:** Pearson's correlations (r) between EHQ scores and implicit body fat and muscularity distortion for various body areas.

	Body fat		Muscularity	
	*r*	*p*	*r*	*p*
Overall body	0.145	0.237	0.179	0.145
Arms and shoulders	0.120	0.331	0.236	0.053
Chest or breast	0.043	0.731	0.209	0.087
Abdomen	0.177	0.148	0.238	0.051
Hips	0.175	0.153	0.162	0.187
Thighs	0.145	0.239	0.137	0.266
Legs	0.139	0.259	0.100	0.416
Most distorted area	0.118^a^	0.351	0.216	0.077

^a^*n* = 64.

### ON and explicit body image distortion (univariate analyses)

Pearson's correlation revealed no association between EHQ scores and explicit body size distortion, *r* = −0.023, *p* = 0.854. The same was observed when Spearman’s correlation was conducted (*r*_*s*_ = 0.024, *p* = 0.848).

### ON and body image (multivariate analysis)

First, in order to reduce the number of variables to include in the multivariate regression, four implicit variables were created: Average Implicit Body fat Dissatisfaction, Average Implicit Muscularity Dissatisfaction, Average Implicit Body Fat Distortion, and Average Implicit Muscularity Distortion. For each of those new variables, the average score from the seven body areas was computed, thus representing average body fat and muscularity dissatisfaction and distortion measures. Table 4 presents reliability indicators (Cronbach alpha) of the four new implicit scales.

**Table 4:** Cronbach alpha—average implicit body fat and muscularity dissatisfaction and distortion.

	Cronbach alpha	Inter-item correlation		
		Mean	Min	Max
Average Implicit Body Fat Dissatisfaction (7 items)	0.928	0.723	0.500	0.974
Average Implicit Muscularity Dissatisfaction (7 items)	0.962	0.796	0.690	0.929
Average Implicit Body Fat Distortion (7 items)	0.899	0.669	0.425	0.956
Average Implicit Muscularity Distortion (7 items)	0.954	0.779	0.572	0.912

When running multivariate regression, only body image measures that correlated a priori with the EHQ were included in the analysis. These criteria were used based on the recommendation of Hosmer and Lemeshow^66^. Also, to ensure parsimony and given the low power of this study, a stepwise approach was used in order to keep only significant variables in the results.

Based on Hosmer and Lemeshow’s^66^ criteria (*p*(r) < 0.250), ten variables were entered in the multivariate regression: Average Implicit Body Fat Dissatisfaction (r = 0.311), Average Implicit Muscularity Dissatisfaction (r = 0.215), Average Implicit Body Fat Distortion (r = 0.160), Average Implicit Muscularity Distortion (r = 0.204), Appearance Evaluation (r = -0.205), Appearance Orientation (r = 0.299), Fitness Orientation (r = 0.339), Health Orientation (r = 0.367), Body Areas Satisfaction (r = −0.235) and Overweight Preoccupation (r = 0.569). Table 5 presents the results of the stepwise regression.

**Table 5:** Multivariate regression predicting EHQ scores. Significant results are indicated in bold characters.

	b (se)	*p*	Beta
Overweight Preoccupation	4.698 (0.803)	**<0.001**	0.528
Health Orientation	5.866 (1.351)	** < 0.001**	0.397
Average Implicit Muscularity Distortion	5.360 (2.271)	**0.021**	0.218
R^2^ = 46.7%			

Among the ten aforementioned variables that were introduced in the stepwise regression, the final regression model identified three predictors that were significantly associated with the EHQ.

Overweight Preoccupation, Health Orientation and Average Implicit Muscularity Distortion showed all positive and significant association with EHQ scores. Overweight Preoccupation presented the largest relative weight (Beta = 0.528) among the final predictors with more than double the weight of the Average Muscularity Distortion (Beta = 0.218) and 33% more than the Health Orientation (Beta = 0.397). The final regression model explained 46.7% of the total variance on the EHQ.

### ON and gender differences

Given the small number of men in the sample (n < 20), a non-parametric test was first conducted to verify if their distribution on the EHQ differed from that of women. An independent samples Mann–Whitney U test did not reveal a significant difference between females and males with respect to the distribution of the EHQ scores (U = 223.500; *p* = 0.446).

Moreover, to verify if the results were maintained without the male sample, the correlation and multiple regression analyses were conducted a second time on women only. There were a few minor differences on univariate correlations, but the same three variables as before came out significant in the multiple regression analysis.

## Discussion

ON, characterised by an extreme focus on healthy eating, is a recently described condition with possibly severe consequences upon physical, social, and psychological levels^3–6^. In the absence of a universally shared definition of the concept of ON, questions have been raised about a possible body image component^6,11^. The present study is the first to include an implicit measure of body image attitudes in relation to ON. To the best of our knowledge, our implicit measure has never been used in the field of body image, and has multiple advantages over existing measures of body image. Ours is also the first study to address body image distortion in relation to ON. Finally, in order to extend the generalization of previous findings, we opted for a less criticized measure of ON^36–39^. and a widely used measure of explicit body image attitudes^63,64^.

### Discussion of results from univariate analyses

#### ON and implicit body image attitudes

Looking solely at correlational analyses, our findings support our hypothesis, and show that the more a person is fixated on healthy eating, the more body fat that person will likely want to lose on their whole body, as well as on their arms and shoulders, chest or breast, abdomen, hips, and thighs. A similar trend was also observed with implicit muscularity dissatisfaction: although the effect sizes were much smaller, ON was related to the desire to increase one’s muscularity on various body parts. As a matter of fact, when both Pearson's and Spearman's correlations were considered, the results indicated that ON may be related with a desire to gain muscularity in the chest or breast, arms and shoulders, hips, and whole body.

#### ON and explicit body image attitudes

Overall, our results derived from correlational analyses support our hypothesis and replicate what has already been found in the literature on ON and explicit body image attitudes. More precisely, they suggest that ON is positively associated with investment in physical health, appearance, and physical fitness, as well as with overweight preoccupation. Such findings are consistent with prior research reporting analogous correlations among various samples^19–21^. Similarly to previously published data, our results did not support an association between ON and fitness evaluation, health evaluation, reactivity to physical illness, and self-classified weight^20,21^. Also, these results only partially support an association between ON and appearance satisfaction. Of general appearance satisfaction and body areas satisfaction measures, only the latter yielded significant results, while both were hypothesized to be associated with ON. Moreover, the body area satisfaction measure only reached significance using Spearman's correlation, while being just above threshold using Pearson's correlation. This contributes to the data disparity surrounding self-report appearance satisfaction in ON literature^19–21,26–29^.

One plausible explanation for the aforementioned contradictory findings regarding appearance satisfaction is that common measures combine various characteristics (e.g. hair, face, height, weight, sexual attractiveness, clothes fitting), which prevent the adequate distinction and measure of relations with more specific elements of appearance dissatisfaction. This may explain why, by using measures that were specific to certain body areas, we were able to reveal a significant correlation between ON and body fat/muscularity dissatisfaction (implicit body image attitudes).

#### ON and body image distortion (implicit and explicit)

The only significant correlation between ON and body muscularity distortion was found in the abdomen area, and suggests that people with high ON symptomatology are more likely to underestimate their abdominal muscularity. This correlation, however, only reached significance using Spearman's correlation, while being just above threshold using Pearson's correlation. Using an all-or-none frame of thought with respect to statistical significance, an approach increasingly criticized in psychology^67^, this might lead us to conclude that ON is not associated with body image distortion. However, it is worth noting that for all body parts, and for both fat and muscularity, all the correlation coefficients indicate the same direction, i.e. a positive correlation between body image distortion and ON. This contrasts with the near-zero correlation suggesting no association between ON and explicit body size distortion.

### ON and body image (multivariate analysis)

Our findings show that, when combining all the aforementioned components of body image, overweight preoccupation, physical health orientation, and average implicit muscularity distortion were independent predictors of higher ON manifestation. That is, explicit concerns and anxiety toward weight and eating is most associated with higher ON symptomatology, followed by investment in having a physically healthy lifestyle, and implicit underestimation of muscularity. Together, those three components accounted for almost half of the variation of ON symptomatology in our sample. Thus, our results suggest that, of all body image components documented here, preoccupation and anxiety about being overweight, investment in physical health, and implicit muscularity underestimation are most associated with ON.

The association between those specific aspects of body image and ON are consistent with previous propositions or findings. First, investment in physical health is in line with the first description of ON, which suggested that extreme focus on healthy eating would arise from a desire to become more healthy or to treat physical illness^3,4^. The association between ON and weight concerns and vigilance, as well as muscularity distortion, also makes sense given that ON is characterized by a fixation on healthy eating^3,4^ on the one hand, and findings suggesting that perception of health is affected by body fat and muscularity^68^ on the other hand. Hence, our results may suggest that people with a fixation on healthy eating may also present a fixation on exhibiting a “healthy ideal body image”, through value of and investment in physical health, fitness, and weight control. Moreover, the distortion regarding self-perceived muscularity is congruent with findings showing an association between ON and exercise addiction and compulsion^23,24^. Nonetheless, these interpretations of our results remain speculative, and more extensive research should be conducted to better understand the mechanisms underlying the association between these specific aspects of body image and ON.

When looking at results from the multivariate analysis, the significant relationship between ON and muscularity distortion only emerged after controlling for overweight preoccupation and health orientation. Interestingly, as discussed earlier, the univariate associations between ON and implicit muscularity distortion for various body areas were mostly not significant, although we consistently found a positive correlation between them. Furthermore, while there was a positive association between health orientation and ON, there was a negative association, albeit non significant, between health orientation and average muscularity distortion (see Table S6 in Supplemental Material). A possible explanation for these results could lie in the idea brought by some researchers who developed the Teruel Orthorexia Scale (TOS), an ON questionnaire differentiating healthy and unhealthy ON^69^. It is in fact possible that health orientation and muscularity distortion are associated with different components of ON, with the former being associated with healthy ON and the latter with unhealthy ON. The present project was not specifically designed to distinguish between these two components of ON, and the TOS was only published after the beginning of our data collection. Nevertheless*, *a posteriori analyses on the different subscales of the EHQ still support the idea that distortion was specifically associated with the pathological component of “interest toward healthy eating”. Table S7 in Supplemental Material shows that the EHQ Problems subscale, measuring problems associated with healthy eating, is the only subscale that correlates with muscularity distortion, while not correlating with health orientation. Still, more extensive research assessing healthy and unhealthy components of ON, should be conducted to specifically disentangle how ON is related to body image distortion.

### Limitations, strengths and future studies

Perhaps the main limitation of this study was its correlational design, as it does not allow to conclude on the directionality of the relation between variables, as well as infer causality. It remains possible that this relationship is reversed or bidirectional. In order to clarify this question, it would be useful to conduct further studies with a longitudinal design.

Likewise, the design used in the present study does not rule out the possibility that another variable not included in our experiments could better explain our results. In the present study, we decided to exclude from our analyses additional measures that were collected as part of a larger research project: screening tools for eating disorders and depression, as well as self-esteem and state anxiety measures (see Supplementary Material for details). The stated aim of this study was to investigate the association between ON symptomatology and body image in a non-clinical sample recruited from the general population and varying in ON symptomatology; we did not intend to reveal criteria that would lead to a differential diagnosis of ON. This decision was markedly based on the knowledge that the aforementioned measures were not developed to provide a differential diagnosis of ON; as such, they may include many items overlapping with the clinical presentation of ON. This is notably the case for the Eating Attitudes Test (EAT-26)^70^, a screening tool for eating disorders that would also be sensitive to ON^71^. Including additional measures as covariables or as exclusion criteria, for instance, based on their score on the EAT-26, would thus have been likely to blur the portrait regarding our main question, namely whether or not varying ON symptomatology in a non-clinical sample is associated with body image. Still, it would be of high interest for future research to conduct moderation analysis to better understand how various variables might modulate the relationship between ON and body image.

Furthermore, sample characteristics such as the disproportionality between males and females and the small number of participants constitute another limitation that is worth mentioning. Although we advertised the study across broad networks, only a small number of men contacted us to take part in it. The number of men was insufficient to perform gender comparisons on the link between ON and body image. We did, however, conduct additional analyses to make sure that the males in our sample were not affecting the pattern of results in a spurious way. Those analyses showed that the data distribution of male participants did not differ significantly from that of female participants in terms of ON symptomatology. Additionally, data from males did not significantly affect the pattern of results in the correlational and multiple regression analyses. Further studies using larger samples should be conducted to document in a more comprehensive manner the relationship between ON and body image. In doing so, special attention should be paid to document gender differences.

Another limitation consists of our decision to consider only implicit attitudes and distortion towards muscularity and body fat. In fact, as mentioned in the first section of this article, the reverse correlation method has the important advantage of not requiring any assumptions regarding the body image dimensions that may be related to ON. This study still offers this advantage, in the sense that participants did not have to think about those specific dimensions during the image-construction phase; thus, the mental representations obtained from our participants were not biased by such assumptions. Yet, in the image-comparison phase, we had to specify a limited number of dimensions on which to compare the photographs and mental representations. Various other dimensions could have been assessed during this phase (e.g. health, attractiveness…), but we decided to focus on muscularity and body fat for two main reasons: it allowed us to compare our results with those of many previous studies, as these two chosen dimensions have frequently been scrutinized in the past ^19–23,25,30,31^, and it was in line with the way ON is conceptualized (i.e. a fixation on healthy eating) as muscularity and body fat are closely linked to the concept of health^68^. Nevertheless, it is plausible that body image is affected in ON in other dimensions not assessed here, especially with regard to perceptions.

Finally, although reverse correlation can be considered as a more implicit measure of distortion and attitudes than self-report questionnaires or other methods requiring participants to assess the size of specific parts of their body, we are aware that the technique is not utterly implicit. The task was not completely orthogonal to the purpose of the study and participants were aware that they were looking at a body. Nevertheless, several participants reported, in the debriefing sessions, not being aware that they were looking at a picture of their own body throughout the experiment.

Despite those limitations, the present study has valuable strengths. First, the multi-method approach provided complementary measures of body image, both implicit and explicit, attitudes as well as distortion, allowing a more comprehensive assessment. Furthermore, we used the EHQ, so as to improve upon existing literature on ON and body image which has mostly made use of the ORTO-15^35^. Moreover, we think that the use of the reverse correlation technique to measure body image not only provides new knowledge on ON, but may also offer a novel tool to researchers in the general field of body image. What makes this technique particularly suitable is that it provides an individualized measure of implicit body image, while still enabling its objective assessment.

The present study has shown how the reverse correlation method might add to current measures of body image, especially in regard to body image distortion. In fact, this method allowed for a unique finding that was not revealed by explicit measures. Even more so, it allowed us to acquire a more precise understanding of what dimension of body image distortion is associated with ON. Indeed, this method provided an insight into how body image distortion, specifically the implicit underestimation of muscularity, is associated with ON severity.

## Conclusion

The present study supports the existence of a link between ON and body image attitudes, both implicit and explicit. For the first time, we show an association between ON symptomatology and body image distortion in a non-clinical sample. Among various attitudinal and perceptual components of body image, explicit overweight preoccupation, explicit physical health investment and implicit muscularity underestimation were most associated with ON symptomatology. Those findings, along with the use of the EHQ as an alternative measure of ON, shed a new light on the relationship between ON and body image, and contribute to the emerging literature on this topic. Since we tested a non-clinical sample, more studies using implicit methods such as reverse correlation, but on individuals presenting more extreme symptoms, could help clarify the question of body image attitudes and distortion in ON.

## Supplementary Information

Supplementary Information.

## Data Availability

The datasets generated during the current study are available from the corresponding author on reasonable request.
