# Supplementary material for: The relationship between orthorexia nervosa symptomatology and body image attitudes and distortion
Source: Sci Rep. 2021 Jun 25;11:13311. doi: 10.1038/s41598-021-92569-2 (PMC8233361; doi:10.1038/s41598-021-92569-2)
Supplement: Supplementary file 1 — Supplementary Information. [file 41598_2021_92569_MOESM1_ESM.docx]

**The Relationship Between Orthorexia Nervosa Symptomatology**

**and Body Image Attitudes and Distortion**

Adrianne Pauzé ^1^, Marie-Pier Plouffe-Demers ^2^, Daniel Fiset ^1^, Dave Saint-Amour ^2^, Caroline Cyr ^1^, & Caroline Blais ^1^*

1. Département de psychoéducation et de psychologie, Université du Québec en Outaouais, Canada

C.P. 1250, succursale Hull, Gatineau (Québec), J8X 3X7, Canada

1. Département de psychologie, Université du Québec à Montréal, Canada

C.P. 8888, succursale Centre-ville, Montréal (Québec), H3C 3P8, Canada

Other authors’ emails (in order of authors): paua07@uqo.ca; plom09@uqo.ca; daniel.fiset@uqo.ca; st-amour.dave@uqam.ca; caroline.cyr.cissso@ssss.gouv.qc.ca.

*Address correspondence to

Caroline Blais, Ph.D.

Département de psychoéducation et de psychologie

Université du Québec en Outaouais

C.P. 1250, Succ. Hull

Gatineau, Qc

J8X 3X7

Phone: 819-595-3900 # 2551

Email: caroline.blais@uqo.ca

**SUPPLEMENTARY MATERIAL**

**1. Additional measures collected measures in the image-construction phase**

The present appendix showcases additional measures that were collected in the image-construction sample in the context of a larger research project, but not discussed in the present study: the validated French translations of the Eating Attitude Test-26 (EAT-26; Garner et al., 1982; Leichner et al., 1994), the Rosenberg Self-Esteem Scale (Rosenberg, 1965; Vallières & Vallerand, 1990), and the Beck Depression Inventory-II (BDI-II; Beck et al., 1998), as well as a French translation of the Subjective Units of Distress Scale (Wolpe, 1969).

**2. Spearman's correlation complementary to main analysis**

Table S1

*Spearman's correlations (r_s_) between EHQ and implicit body fat or muscularity dissatisfaction for various body areas.*

| Body areas | Body fat | | Muscularity | |
| --- | --- | --- | --- | --- |
|  | *r_s_* | *p* | *r_s_* | *p* |
| Overall body | **0.331** | **0.006** | **0.257** | **0.034** |
| Arms and shoulders | **0.256** | **0.035** | **0.260** | **0.032** |
| Chest or breast | **0.258** | **0.034** | **0.284** | **0.019** |
| Abdomen | **0.281** | **0.020** | 0.225 | 0.065 |
| Hips | **0.316** | **0.009** | **0.283** | **0.019** |
| Thighs | **0.265** | **0.029** | 0.144 | 0.241 |
| Legs | 0.184 | 0.133 | 0.211 | 0.085 |
| Most unsatisfactory area | **0.295^a^** | **0.017** | **0.251^b^** | **0.041** |

*^a^ n = 65. ^b^ n = 67.*

Table S2

*Spearman's correlations (r_s_) between EHQ and MBSRQ subscales.*

| MBSRQ subscales | EHQ | |
| --- | --- | --- |
|  | *r_s_* | *p* |
| Appearance Evaluation | -0.219 | 0.073 |
| Appearance Orientation | 0.224 | 0.067 |
| Fitness Evaluation | 0.017 | 0.888 |
| Fitness Orientation | **0.353** | **0.003** |
| Health Evaluation | -0.036 | 0.771 |
| Health Orientation | **0.425** | **<0.001** |
| Illness Orientation | 0.125 | 0.309 |
| Body Areas Satisfaction | **-0.244** | **0.045** |
| Overweight Preoccupation | **0.528** | **<0.001** |
| Self-Classified Weight | 0.148 | 0.230 |

Table S3

*Spearman's correlations (r_s_) between EHQ and implicit body fat and muscularity distortion for various body areas.*

| Body areas | Body fat | | Muscularity | |
| --- | --- | --- | --- | --- |
|  | *r_s_* | *p* | *r_s_* | *p* |
| Overall body | 0.155 | 0.207 | 0.162 | 0.187 |
| Arms and shoulders | 0.087 | 0.483 | 0.182 | 0.137 |
| Chest or breasts | 0.002 | 0.989 | 0.151 | 0.219 |
| Abdomen | 0.154 | 0.209 | **0.248** | **0.041** |
| Hips | 0.167 | 0.172 | 0.120 | 0.329 |
| Thighs | 0.181 | 0.139 | 0.089 | 0.471 |
| Legs | 0.141 | 0.250 | 0.059 | 0.635 |
| Most distorted area | 0.149^a^ | 0.239 | 0.215 | 0.078 |

^a^ *n* = 64.

**3. Descriptive Statistics**

Table S4

*Descriptive statistics for Eating Habit Questionnaire (EHQ), Multidimensional Body-Self Relations Questionnaire (MBSRQ) subscales and Explicit Body Size Distortion.*

| Measures | Mean *(SD)* | [Lowest score, Highest score] |
| --- | --- | --- |
| EHQ | 40.44 (8.06) | [27, 68] |
| MBSRQ Subscales |  |  |
| Appearance Evaluation | 3.56 (0.70) | [1.57, 5.00] |
| Appearance Orientation | 3.33 (0.59) | [2.17, 4.42] |
| Fitness Evaluation | 3.71 (0.95) | [1.33, 5.00] |
| Fitness Orientation | 3.52 (0.85) | [1.62, 5.00] |
| Health Evaluation | 3.71 (0.71) | [1.83, 5.00] |
| Health Orientation | 3.38 (0.55) | [2.25, 4.50] |
| Illness Orientation | 3.04 (0.68) | [1.80, 4.80] |
| Body Area Satisfaction | 3.42 (0.71) | [1.33, 5.00] |
| Overweight Preoccupation | 2.44 (0.91) | [1.33, 5.00] |
| Self-Classified Weight | 3.23 (0.68) | [1.50, 5.00] |
| Explicit Body Size Distortion | -0.08 (0.56) | [-3.27, 0.80] |

*Note*. Theoretical range for EHQ scores is 21 to 84. Theoretical range for MBSRQ subscales scores are 1 to 5. Theoretical range for Explicit Body Size Distortion scores is -4 to +4.

Table S5

*Descriptive statistics for implicit body fat and muscularity dissatisfaction and distortion for various body areas and average score.*

| Body areas | Body image dissatisfaction | | Body image distortion | |
| --- | --- | --- | --- | --- |
|  | Body fat | Muscularity | Body fat | Muscularity |
| Overall body | 0.37 (0.46)  [-0.38, 1.88] | 0.16 (0.45)  [-0.63, 1.37] | 0.02 (0.34)  [-1.09, 0.88] | 0.60 (0.43)  [-0.50, 1.38] |
| Arms/shoulders | 0.13 (0.22)  [-0.34, 0.81] | -0.02 (0.37)  [-0.83, 1.33] | 0.01 (0.13)  [-0.28, 0.50] | 0.51 (0.29)  [-0.03, 1.13] |
| Chest/breasts | 0.15 (0.32)  [-0.72, 1.00] | 0.06 (0.42)  [-0.80, 1.53] | -0.05 (0.24)  [-0.91, 0.50] | 0.42 (0.36)  [0.24, 1.17] |
| Abdomen | 0.45 (0.55)  [-0.53, 1.97] | 0.28 (0.54)  [-0.72, 1.57] | 0.04 (0.44)  [-1.25, 1.28] | 0.62 (0.50)  [-0.83, 1.62] |
| Hips | 0.26 (0.34)  [-0.38, 1.22] | 0.01 (0.38)  [-0.63, 0.86] | 0.04 (0.21)  [-0.59, 0.59] | 0.43 (0.33)  [-0.48, 1.03] |
| Thighs | 0.14 (0.26)  [-0.34, 0.75] | -0.09 (0.40)  [-0.80, 0.80] | -0.02 (0.18)  [-0.44, 0.50] | 0.50 (0.32)  [-0.52, 1.10] |
| Legs | 0.07 (0.14)  [-0.19, 0.56] | -0.07 (0.37)  [-0.73, 1.03] | 0.00 (0.08)  [-0.22, 0.19] | 0.52 (0.32)  [-0.41, 1.33] |
| Most unsatisfactory or distorted area | 0.49 (0.58)  [-0.72, 1.97]^a^ | 0.17 (0.69)  [-0.83, 1.57]^b^ | 0.06 (0.48)  [-1.25, 1.28]^c^ | 0.73 (0.46)  [-.083, 1.62] |
| Average score | 0.23 (0.30)  [-0.28, 0.96] | 0.05 (0.38)  [-0.71, 1.20] | 0.01 (0.20)  [-0.67, 0.59] | 0.51 (0.33)  [-0.31, 1.09] |

*Note*. Mean, standard deviation in parentheses, lowest and highest scores obtained in brackets. Theoretical range for Reverse Correlation technique scores is -3 to +3. ^a^ *n* = 65. ^b^ *n* = 67. ^c^ *n* = 64.

**4. Implicit vs. Explicit body image**

The relation between implicit and explicit body image was assessed using Pearson's and Spearman's correlations between Average Implicit body Fat and Muscularity Dissatisfaction and Distortion scores from the reverse correlation method and either MBSRQ subscales (for body image attitudes) or Explicit Body Size Distortion (for body image perceptions). Spearman’s analyses showed similar coefficients, so results are not presented here. Pearson’s correlation scores are presented in Table S6.

Analyses revealed significant negative correlations between Average Implicit Body Fat Dissatisfaction and Appearance Evaluation, Body Area Satisfaction, Health Evaluation. Results also showed significant positive correlations with Overweight Preoccupation and Self-Classified Weight. However, there was no association between Average Implicit Body Fat Dissatisfaction scores and Orientation subscales (appearance, fitness, health, and illness), as well as with Fitness Evaluation.

We obtained significant negative correlations between Average Implicit Muscularity Dissatisfaction and Appearance Evaluation, Body Area Satisfaction. We also found significant positive correlations with Self-Classified Weight. However, there was no association between Average Implicit Muscularity Dissatisfaction and Orientation subscales (appearance, fitness, health, and illness), nor with Fitness Evaluation or Overweight Preoccupation.

As for body image perception, results showed a significant correlation between Explicit Body Size Distortion and Average Implicit Muscularity Distortion. However, the association was slightly above the significant threshold for Implicit Body Fat Distortion, although there was also a positive tendency.

Table S6

*Pearson's correlation (and p values) between MBSRQ (explicit attitudes), Explicit Body Size Distortion, and Average Implicit Muscularity and Body Fat Dissatisfaction and Distortion.*

| Explicit body image measures | Average Implicit Body Fat Dissatisfaction | | Average Implicit Muscularity  Dissatisfaction | | Average Implicit Body Fat Distortion | | Average Implicit Muscularity  Distortion | |
| --- | --- | --- | --- | --- | --- | --- | --- | --- |
|  | *r* | *p* | *r* | *p* | *r* | *p* | *r* | *p* |
| MBSRQ subscales |  |  |  |  |  |  |  |  |
| Appearance Evaluation | **-0.540** | **<0.001** | **-0.276** | **0.023** | -0.184 | 0.133 | -0.161 | 0.191 |
| Appearance Orientation | 0.045 | 0.713 | 0.186 | 0.128 | 0.086 | 0.485 | 0.179 | 0.144 |
| Fitness Evaluation | -0.209 | 0.087 | 0.018 | 0.886 | -0.105 | 0.396 | -0.142 | 0.249 |
| Fitness Orientation | -0.104 | 0.398 | 0.002 | 0.986 | -0.009 | 0.940 | 0.054 | 0.662 |
| Health Evaluation | **-0.339** | **0.005** | 0.226 | 0.064 | -0.223 | 0.067 | -0.214 | 0.079 |
| Health Orientation | - 0.055 | 0.657 | 0.101 | 0.411 | -0.214 | 0.080 | -0.214 | 0.079 |
| Illness Orientation | -0.127 | 0.301 | 0.103 | 0.403 | -0.160 | 0.192 | -0.050 | 0.686 |
| Body Areas Satisfaction | **-0.456** | **<0.001** | **-0.301** | **0.013** | -0.163 | 0.185 | -0.156 | 0.204 |
| Overweight Preoccupation | **0.378** | **0.001** | 0.221 | 0.070 | 0.115 | 0.352 | 0.135 | 0.272 |
| Self-Classified Weight | **0.435** | **<0.001** | **0.311** | **0.010** | 0.080 | 0.517 | -0.084 | 0.498 |
| Explicit Body Size Distortion | -0.102 | 0.407 | 0.065 | 0.599 | 0.228 | 0.062 | **0.318** | **0.008** |

**5. A posteriori analysis with EHQ subscales**

Specific a posteriori analysis was conducted to better understand the apparent disparity between univariate and multivariate analysis in regard to implicit muscularity distortion and health orientation. Pearson’s correlation was thus conducted with those two components of body image and the three subscales of ON: Problems (problems associated with healthy eating), Knowledge (healthy eating knowledge and behaviors), and Feelings (feeling positively about healthy eating).

As shown in Table S7, significant positive associations were found between EHQ Problems subscale and Average Implicit Muscularity Distortion, and between Health Orientation and EHQ Feeling subscale.

Table S7

*Pearson's correlation (r) between Average Implicit Muscularity Distortion (score from reverse correlation method), and Health Orientation (a MBSRQ sbscale).*

| EHQ subscales | Average Implicit Muscularity Distortion | | Health Orientation | |
| --- | --- | --- | --- | --- |
|  | *r* | *p* | *r* | *p* |
| Problems | **0.250** | **0.039** | 0.184 | 0.134 |
| Knowledge | 0.149 | 0.224 | 0.147 | 0.232 |
| Feeling | 0.027 | 0.828 | **0.548** | **<0.001** |
